# Improving Patient Prioritization During Hospital-Homecare Transition: Protocol for a Mixed Methods Study of a Clinical Decision Support Tool Implementation

**DOI:** 10.2196/20184

**Published:** 2021-01-22

**Authors:** Maryam Zolnoori, Margaret V McDonald, Yolanda Barrón, Kenrick Cato, Paulina Sockolow, Sridevi Sridharan, Nicole Onorato, Kathryn Bowles, Maxim Topaz

**Affiliations:** 1 School of Nursing Columbia University New York, NY United States; 2 Center for Home Care Policy & Research Visiting Nurse Service of New York New York, NY United States; 3 College of Nursing and Health Professions Drexel University Drexel, NY United States; 4 School of Nursing University of Pennsylvania Philadelphia, NY United States

**Keywords:** clinical decision support system, homecare agencies, rehospitalization, RE-AIM framework, PREVENT, effective implementation

## Abstract

**Background:**

Homecare settings across the United States provide care to more than 5 million patients every year. About one in five homecare patients are rehospitalized during the homecare episode, with up to two-thirds of these rehospitalizations occurring within the first 2 weeks of services. Timely allocation of homecare services might prevent a significant portion of these rehospitalizations. The first homecare nursing visit is one of the most critical steps of the homecare episode. This visit includes an assessment of the patient’s capacity for self-care, medication reconciliation, an examination of the home environment, and a discussion regarding whether a caregiver is present. Hence, appropriate timing of the first visit is crucial, especially for patients with urgent health care needs. However, nurses often have limited and inaccurate information about incoming patients, and patient priority decisions vary significantly between nurses. We developed an innovative decision support tool called *Priority for the First Nursing Visit Tool* (PREVENT) to assist nurses in prioritizing patients in need of immediate first homecare nursing visits.

**Objective:**

This study aims to evaluate the effectiveness of the PREVENT tool on process and patient outcomes and to examine the reach, adoption, and implementation of PREVENT.

**Methods:**

Employing a pre-post design, survival analysis, and logistic regression with propensity score matching analysis, we will test the following hypotheses: compared with not using the tool in the preintervention phase, when homecare clinicians use the PREVENT tool, high-risk patients in the intervention phase will (1) receive more timely first homecare visits and (2) have decreased incidence of rehospitalization and have decreased emergency department use within 60 days. Reach, adoption, and implementation will be assessed using mixed methods including homecare admission staff interviews, think-aloud observations, and analysis of staffing and other relevant data.

**Results:**

The study research protocol was approved by the institutional review board in October 2019. PREVENT is currently being integrated into the electronic health records at the participating study sites. Data collection is planned to start in early 2021.

**Conclusions:**

Mixed methods will enable us to gain an in-depth understanding of the complex socio-technological aspects of the hospital to homecare transition. The results have the potential to (1) influence the standardization and individualization of nurse decision making through the use of cutting-edge technology and (2) improve patient outcomes in the understudied homecare setting.

**Trial Registration:**

ClinicalTrials.gov NCT04136951; https://clinicaltrials.gov/ct2/show/NCT04136951

**International Registered Report Identifier (IRRID):**

PRR1-10.2196/20184

## Introduction

### Background

Each year, more than 5 million patients are admitted to the approximately 12,000 homecare agencies across the United States [1-3]. Registered nurses (RNs) make several critical decisions before and during homecare admission, including identifying patients at risk for poor outcomes who might benefit from early interventions. However, there are no rigorously developed standards to assist in making these important decisions.

In this study, we focus on patients admitted to homecare from hospitals because their recent acute exacerbation makes them likely to be in urgent need of timely care, and they constitute up to 70% of all homecare recipients [3,4]. Nationwide evidence shows that about 20% of homecare patients are rehospitalized during the up to 60-day homecare episode [1,5], with as many as 68% of these rehospitalizations occurring within the first 2 weeks of services [4,6-9]. The Medicare regulations require a visit within 48 hours of referral to homecare, but for many patients, this may be too late. A growing body of evidence shows that a significant portion of rehospitalizations may be prevented by timely and appropriately targeted homecare services [4,8,10,11]. Patient prioritization at the time of transition to homecare services is a critical key to better patient outcomes.

The first homecare visit, usually conducted by an RN, is one of the most critical steps of the homecare episode [12,13]. This start of care visit includes an assessment of the patient’s capacity for self-care, medication reconciliation, an examination of the home environment, and a discussion regarding whether a caregiver is present and able to help. A unique care plan is created based on this evaluation of the patient’s needs [14]. Hence, appropriate timing of the first visit is crucial, especially for patients with urgent health care needs. However, recent research by our team showed that nurses have very limited and often inaccurate information about incoming patients due to lack of information exchanges between care settings and lack of standards about the necessary information needed for patient prioritization [12-15]. Often operating with limited information, resources, and time [13], nurses must decide how to prioritize the patient’s first homecare visit.

Some studies have examined solutions for improving patient prioritization in a postacute care setting. For example, one study from Canada developed a method called Method for Assigning Priority Levels (MAPLe) to assist case managers in determining the relative priority that should be attached to patients when postacute care referrals are made [16]. However, the MAPLe system is not specific to homecare, and it prioritizes patients irrespective of the care setting. Another study from the United States has developed a tool that helps prioritize patients discharged to skilled nursing facilities [17]. None of the existing studies have developed prioritization tools specific to homecare.

### Objectives

Our team has developed an innovative clinical decision support system (CDSS) called *Priority for the First Nursing Visit Tool* (PREVENT) to assist nurses in prioritizing patients in need of immediate first homecare nursing visits [18]. PREVENT was developed with rigor, using a strong theoretical foundation (transition theory) [19] and methodology for eliciting experts’ decisions to create clinical decision support tools [20]. PREVENT was constructed using data mining, regression modeling, and expert homecare nurses’ ratings of example patients who were transitioned from hospital to homecare. The goal was to identify key patient characteristics that are essential to support early homecare admission decision making. Overall, more than 70 patient demographic and clinical characteristics (eg, comorbidities, level and availability of social support, and detailed functional status) were considered for inclusion in the final prediction model from which PREVENT was developed. The final PREVENT CDSS uses 5 factors (including the number of medications, number of comorbid conditions, presence of a wound, presence of a comorbid condition of depression, and patient’s functional status) to produce a recommendation on whether a specific homecare patient should be prioritized for the first homecare nursing visit. See Multimedia Appendix 1 [16-18,21-23] for more information about the methods for PREVENT CDSS development.

We completed a pilot efficacy study [9] to measure the efficacy of PREVENT, conducted at a large urban hospital in Brooklyn, New York. In collaboration with the Visiting Nurse Service of New York (VNSNY), we enrolled 176 patients admitted to homecare from the hospital during April and May 2016. In the control phase (n=90 patients), we calculated the PREVENT priority score but did not share the score with the homecare admission staff who influence visit scheduling. In the experimental phase, the PREVENT score was shared with the homecare admission staff (n=86 patients). During this phase, patients identified as high priority received their first homecare nursing visit about a half-day sooner as compared with the control phase (1.8 days vs 2.2 days; *P*=.09). Rehospitalizations from homecare decreased by almost 50% (9.4% point reduction) when comparing the control (21.1%) and experimental phases (11.7%), with a significant difference between the rehospitalization (survival analysis) curves (log-rank *P* =.03). We acknowledge that this pilot study had a relatively small sample size and potentially insufficient adjustment for background variables. However, these results were promising in that high-priority patients received their first homecare visit sooner and overall rehospitalization rates were lower.

This manuscript presents our methodology for examining PREVENT’s impact on patient outcomes via a larger and more rigorous effectiveness trial. Our specific study aims are as follows:

1. Evaluate the effectiveness of the PREVENT tool on process and patient outcomes. We will test the following hypotheses using survival analysis and logistic regression with propensity score matching: compared with not using the tool in the preintervention phase, when homecare clinicians use the PREVENT tool, high-risk patients in the intervention phase will receive more timely first homecare visits and have decreased incidence of rehospitalization and have decreased emergency department use within 60 days of hospital discharge.
2. Explore PREVENT’s reach and adoption by the homecare admission staff and describe the tool’s implementation during homecare admission. Aim 2 will be assessed using mixed methods incorporating homecare admission staff interviews, think-aloud simulations [24], and analysis of staffing and other relevant data.

## Methods

### Mixed Method Approach

We are using an embedded mixed methods design. We will conduct a pre- and postintervention trial of PREVENT’s integration into clinical practice using homecare admissions from two New York City urban hospitals serving diverse racial and ethnic populations. We will use quantitative methods, including logistic regression and survival analysis, to evaluate the effects of the tool on process and patient outcomes. We will utilize qualitative methods integrated with quantitative methods to gain an in-depth insight into technology adoption and implementation.

### Setting

On the basis of our consultations with New York-Presbyterian (NYP) hospitals’ leadership and our goal of exploring the effectiveness of the PREVENT system in different settings and among sites serving an ethnically diverse population, we will conduct the study at 2 NYP hospitals: (1) NYP Hospital/Columbia University Irving Medical Center (large academic medical center), a 745-bed adult academic medical center providing emergency, primary, and specialty care in all the major fields of medicine, and (2) NYP Allen Hospital (small community hospital), a 196-bed community hospital serving northern Manhattan, Riverdale, and other communities in the Bronx. As a homecare site, we will use VNSNY—the largest not-for-profit home health agency in the United States serving up to 48,500 patients and health plan members daily.

### Conceptual Model

The study is guided by the Reach, Effectiveness, Adoption, Implementation, and Maintenance (RE-AIM) framework. The RE-AIM was adopted [25] to specifically focus on real-world implementation of clinical informatics interventions, such as CDSS. Since its adoption, RE-AIM has been successfully applied in numerous studies of health information technology evaluations [26]. The components of this framework are as follows: *reach* of the intervention to a representative proportion of the target population, *effectiveness* of the intervention, *adoption* of the intervention across a broad and representative proportion of settings, *implementation* details, and *maintenance* of the intervention after implementation. In each phase of implementation, we will use the mixed methods approach for data collection and analysis to identify barriers and facilitators of the PREVENT implementation. Table 1 summarizes the RE-AIM dimensions with the associated definitions of each component, questions that need to be addressed in each component, and the associated analytic methods to answer those questions.

**Table 1:** Reach, Effectiveness, Adoption, Implementation, and Maintenance framework dimensions in this study.

RE-AIM^a^ dimension and definition	Questions relevant to this study and aims addressed	Analytic methods	Data sources
Reach (individual level): Absolute number, proportion, and representativeness of individuals who participate in the intervention.	1. Were patients for whom PREVENT^b^ was calculated representative of all the eligible patients? (*Aim 2*)	*Quantitative analysis*: Comparison of patient demographic and clinical characteristics within the study (preintervention vs intervention groups), and between the study period and annual data (method: *t* tests or chi-square tests, when applicable).	VNSNY^c^ data repository (including OASIS^d^) [27]
Individuals who participate in the intervention.	2. What proportion of admission staff eligible to use the PREVENT actually used it? (*Aim 2*)	*Quantitative analysis*: Estimation of how many admission staff used PREVENT out of total eligible admission staff members.	Data from VNSNY admission units
Efficacy or effectiveness (individual level): Impact of an intervention on important outcomes, including potential negative effects.	3. What is the effect of PREVENT on process outcomes? (*Aim 1*)	*Quantitative analysis*: Estimation of PREVENT’s effect on timing of the first homecare visit (method: survival analysis).	VNSNY data repository (including OASIS)
Efficacy or effectiveness (individual level)	4. What is the effect of PREVENT on patient outcomes? (*Aim 1*)	*Quantitative analysis*: Estimation of PREVENT’s effect on incidence and time to rehospitalization and ED^e^ use within 30 and 60 days (method: logistic regression and survival analysis).	VNSNY data repository (including OASIS) +RHIO^f^ (health service use data in New York)
Adoption (setting and/or organizational level): Absolute number, proportion, and representativeness of settings and intervention agents (people who deliver the program) who are willing to initiate a program.	5. What are the characteristics of the settings that decided to adopt PREVENT? (*Aim 2*)	Quantitative analysis: Description of the VNSNY and two referring hospitals in terms of location, staffing, and patient population (method: descriptive summary).	VNSNY staffing data and hospitals staffing data; VNSNY data repository
Adoption (setting and/or organizational level)	6. How well did the goals of PREVENT fit with the values and expectations of the practice settings? (*Aim 2*)	Qualitative analysis: Identification of the strategic plans and potential other incentives (such as CMS^g^ readmission reduction program) related to PREVENT (method: descriptive summary).	VNSNY and NYP^h^ strategic plans; information about potential other incentives
Implementation (setting and/or organizational level): Setting level—intervention agents’ fidelity to the various elements of an intervention’s protocol, including consistency of delivery as intended and the time and cost of the intervention; individual level—clients’ use of the intervention strategies.	7. How many admission staff members used PREVENT? (*Aim 2*)	Quantitative analysis: Description of the number of admission staff members who used PREVENT (method: descriptive statistics).	VNSNY data repository
Implementation (setting and/or organizational level)	8. What was the user satisfaction with PREVENT? (*Aim 2*)	Quantitative analysis (method: summary statistics).	Administration of the 12 item End-User Computing Satisfaction Instrument
Implementation (setting and/or organizational level)	9. Did users perceive PREVENT as easy to use? (*Aim 2*)	Qualitative analysis (method: thematic analysis).	Postintervention interviews
Implementation (setting and/or organizational level)	10. Did users perceive PREVENT as useful? (*Aim 2*)	Qualitative analysis (method: thematic analysis).	Postintervention interviews
Implementation (setting and/or organizational level)	11. Was there sufficient leadership support? (*Aim 2*)	Qualitative analysis (method: thematic analysis).	Administration of open-ended questions during think-aloud simulations and postintervention interviews
Implementation (setting and/or organizational level)	12. What workflow adjustments needed to be made to streamline PREVENT into routines of daily clinical practice? (*Aim 2*)	Qualitative analysis (method: thematic analysis).	Research team experience; administration of open-ended questions during think-aloud simulations and postintervention interviews
Implementation (setting and/or organizational level)	13. What support, resources, and outside collaborations were needed to implement PREVENT? (*Aim 2*)	Qualitative analysis (method: thematic analysis).	Research team experience; agency description of PREVENT electronic integration process
Implementation (setting and/or organizational level)	14. What technical infrastructure was required to implement the CDSS^i^? (*Aim 2*)	Qualitative analysis (method: thematic analysis).	Research team experience; agency description of PREVENT electronic integration process
Implementation (setting and/or organizational level)	15. What user training and support services were needed by PREVENT users? (*Aim 2*)	Qualitative analysis (method: thematic analysis).	Research team experience; data from think-aloud simulations and postintervention interviews
Implementation (setting and/or organizational level)	16. What were the potential barriers to successful PREVENT implementation and how were they addressed? (*Aim 2*)	Qualitative analysis (method: thematic analysis).	Research team experience; administration of open-ended questions during think-aloud simulations and postintervention interviews
Implementation (setting and/or organizational level)	17. Did homecare admission staff agree with PREVENT recommendations? (*Aim 2*)	Quantitative analysis: Comparison of PREVENT recommendations with actual homecare admission staff decisions to provide priority visits and comparison of patient characteristics between cases that disagree versus agree with PREVENT recommendations (method: *t* tests or chi-square tests, when applicable).	Service use and patient data from VNSNY data repository
Implementation (setting and/or organizational level)	18. Did homecare admission staff agree with PREVENT recommendations? (*Aim 2*)	Qualitative analysis (method: thematic analysis).	Administration of open-ended questions during think-aloud simulations and postintervention interviews and follow-up calls to assess disagreements
Maintenance: Setting level—extent to which intervention becomes institutionalized or part of the routine organizational practices; individual level—long-term effects of a program on outcomes for 6 or more months after the most recent intervention contact.	Beyond the scope of this study	Beyond the scope of this study	Beyond the scope of this study

^a^RE-AIM: Reach, Effectiveness, Adoption, Implementation, and Maintenance.

^b^PREVENT: Priority for the First Nursing Visit Tool.

^c^VNSNY: Visiting Nurse Service of New York.

^d^OASIS: Outcome and Assessment Information Set.

^e^ED: emergency department.

^f^RHIO: Regional Health Information Organization.

^g^CMS: Centers for Medicare & Medicaid Services

^h^NYP: New York-Presbyterian.

^i^CDSS: clinical decision support system.

### Study Intervention: PREVENT

The PREVENT tool will be integrated with the hospitals’ electronic health record (EHR) via a locally developed system called iNYP, which integrates with the EHR and provides advanced data review capabilities of all EHR data. iNYP is a Java-based service-oriented web app that builds on Columbia University’s 25-year history of clinical information system innovation [28,29]. iNYP is available as a custom tab within the commercial hospital EHR (supplementing the native results review capabilities) and is also accessible from a web browser or a mobile device. iNYP is widely used by most clinicians alongside the EHR, including the homecare admission staff. The PREVENT score will be calculated automatically from EHR data that populate the patient discharge summary or other parts of the EHR. We have cross-mapped the elements (eg, number of medications and comorbid conditions) needed for the calculation of the PREVENT score to confirm that the required elements are readily available in the EHR system. VNSNY admission staff will receive an auto-populated field within the homecare referral containing the PREVENT recommendation about visit priority, presented as high priority and medium or low priority. Before any data collection, we will test the accuracy of the PREVENT score on the first 50 priority calculations and correct the EHR integration if any mistakes are found.

### Standard VNSNY Patient Admission Workflow

During our preliminary work, we determined that the scheduling and assignment unit assumes responsibility for patient admission to the VNSNY. The unit comprises several admission staff members who are involved in the admission processes, including intake coordinators, clinical associate managers, and schedulers. Homecare admission starts with standard homecare referral signed by the referring physician.

The referrals are passed to the intake coordinators (administrative staff) who enter the referral information into the VNSNY EHR system. Next, clinical field managers give patients a welcome call and coordinate the general start of care dates. After that, schedulers identify the date of a first homecare nursing visit. Each geographic location (based on city boroughs and street addresses) is served by several admission staff members.

### Study Workflow

**Figure 1:**
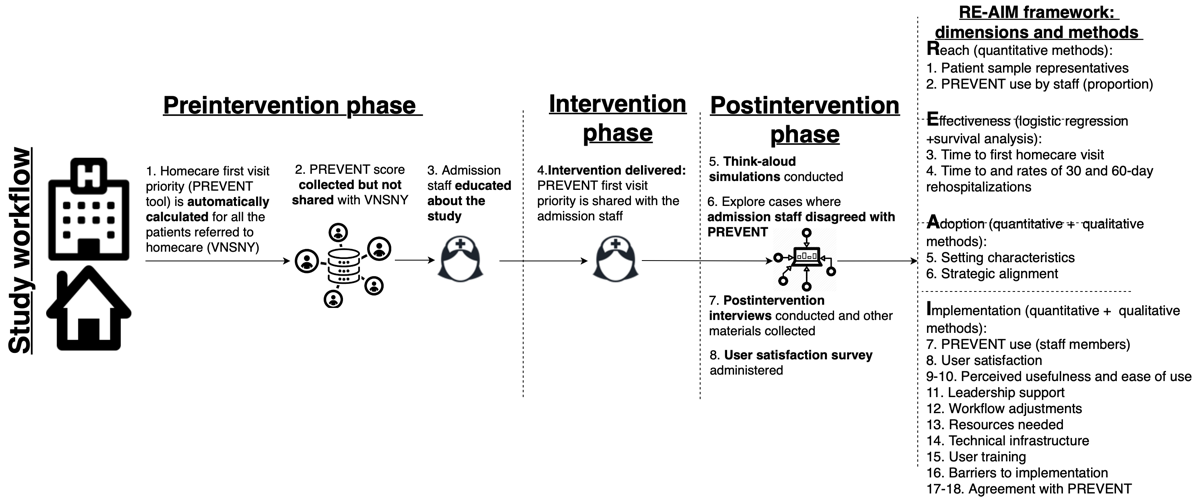
Study workflow and design. PREVENT: Priority for the First Nursing Visit Tool; RE-AIM: Reach, Effective-ness, Adoption, Implementation, and Maintenance; VNSNY: Visiting Nurse Service of New York.

The workflow of PREVENT implementation consists of 3 phases: preintervention phase, intervention phase, and postintervention phase. Figure 1 provides an overview of these phases.

#### Preintervention Phase

During this phase of the study, 3 research activities will be implemented. First, the PREVENT priority score will be automatically calculated for all the patients referred to VNSNY from the 2 hospitals (step 1). Second, the PREVENT score (and priority recommendation based on the score) will be collected but not shared with homecare admission staff over about 3 months (see the *Sample Size Calculation* section; step 2). Third, after the preintervention phase data are collected, the study team will conduct several 30-min educational sessions for the admission staff about the development and validation of PREVENT and this study (step 3). We will work with the VNSNY scheduling and assignment unit management to identify all the VNSNY staff eligible (15-20 staff) to be exposed to PREVENT’s recommendations during the study. We will ensure that each eligible admission staff member undergoes at least one educational session about the study workflow.

#### Intervention Phase

To minimize periodical and time effects, the intervention phase will start at both hospitals on the same date. The PREVENT recommendation will be shared with the homecare intake coordinators for about 3 months (step 4). The intake coordinator will enter the PREVENT recommendation into the *special recommendations* field of the VNSNY EHR system. This field stores information about any special programs or services patients should receive in homecare, such as recommendations for frontloading of visits. Next, clinical field managers and schedulers will incorporate the PREVENT priority recommendations in their processes related to visit scheduling and patient prioritization. The field clinician will then conduct the first nursing visit. For cases where patient prioritization was not possible, we will ask the admission staff to document why a priority visit could not happen (such as the patient refused or short staffing; step 5).

#### Postintervention Phase

After completion of data collection, we will continue to share the results of the PREVENT recommendation for approximately 4-6 weeks. During this period, four evaluation activities will be conducted. First, we will use the *Postintervention simulation guide* (see *Study Instruments* section) to conduct think-aloud simulations with admission staff who were exposed to the CDSS (step 6). Second, concurrently with the think-aloud simulations, we will continuously assess cases where admission staff did not implement PREVENT recommendations (step 7). We will compare patient characteristics between cases that disagreed versus agreed with PREVENT recommendations. We want to identify additional factors that PREVENT might have missed when assessing a patient’s priority for a visit. Once saturation in responses is achieved and we have a solid number of cases for quantitative disagreement comparisons (about 100 cases of disagreement), we will discontinue sharing the PREVENT recommendation. Third, we will anonymously distribute the End-User Computing Satisfaction Instrument using a secure web-based survey platform (Qualtrics [30]; step 8). Fourth, we will conduct in-person interviews using the *Postintervention phase interview guide* with the same respondents who participated in the observations (step 9).

### Study Instruments

Qualitative interviews and think-aloud simulations will be guided by two robust interview guides we will develop for this study. The guides will incorporate aspects of the RE-AIM framework dimensions as questions. The guides are as follows: (1) postintervention simulation guide (think-aloud protocol) and (2) postintervention phase interview guide. Each interview guide will include semi-structured open-ended questions to be answered by the admission staff. The *Postintervention phase interview guide* will include questions about PREVENT’s perceived usability and ease of use, leadership support, workflow adjustments, adequacy of training sessions, and barriers to implementation such as any changes the respondent made to his or her regular workflow to use PREVENT’s recommendations.

The End-User Computing Satisfaction Instrument [31-33] will be used to quantitatively measure satisfaction. The 12 item instrument measures concepts such as accuracy and ease of use and has been used to evaluate many types of applications, including decision support. A score of 54 corresponds to the 70th percentile. Any concept scoring less than the 70th percentile from either user group will guide future tool revision. See Multimedia Appendix 2 [31-33] for more information on the study instruments.

### Methods for Study Aim 1: Evaluate the Effects of PREVENT on Process and Patient Outcomes

#### Sample Size Calculation

In this study, we will calculate the PREVENT scores for all patients referred to VNSNY from the 2 hospitals (see study setting) in a 3 month period during the preintervention phase (scores not shared) and a 3 month intervention phase (scores shared) for an estimated total of 2094 patients and 1508 high-priority patients, respectively. This calculation is based on the pilot study rehospitalization decrease. Multimedia Appendix 3 presents the minimum detectable difference between high-priority intervention group patients and preintervention high-priority patients.

#### Data Sources

Patient characteristics will be extracted from the Outcome and Assessment Information Set (OASIS), a standardized assessment tool designed to collect nearly 100 items related to a recipient’s functional status, clinical status, and service needs during a homecare episode. Mandated by CMS since 1999, OASIS is the most comprehensive national data set for homecare patient assessment and outcomes. Data were collected upon admission, every 60 days, if transferred to an inpatient facility, and at discharge. OASIS data will be extracted from the VNSNY EHR and supplemented with additional data such as language spoken and residence county. Table 2 shows the details of the variables and the sources of the data.

The first visit timing will be extracted from the VNSNY administrative database. In this study, we will examine the impact of PREVENT’s recommendation on the time from the patient’s hospital discharge to the first homecare nursing visit.

**Table 2:** Conceptual domains, measures, and data sources.

Domain	Measures	Data sources
Socio-economic factors	Age, sex, race and ethnicity, language, caregiver support, living arrangements, language spoken, and residence county	OASIS^a^/VNSNY^b^ EHR^c^
Clinical factors	Primary and background diagnoses, limitations in functioning, cognitive status, depressive symptoms, behavioral problems, wounds, pain, sensory status, elimination status, and medications	OASIS/VNSNY EHR
Health outcomes	Time from hospital discharge to first homecare visit, rehospitalizations, and ED^d^ visits	Regional Health Information organization/ VNSNY EHR

^a^OASIS: Outcome and Assessment Information Set.

^b^VNSNY: Visiting Nurse Service of New York.

^c^EHR: electronic health record.

^d^ED: emergency department.

#### Rehospitalization Data

Most often when patients are rehospitalized, they return to the same facility as the initial hospitalization. In this study, it would be the NYP system. We will have access to these data. See Multimedia Appendix 4 for more information about rehospitalization data.

#### Quantitative Analysis Methods

We will use survival analysis methods and logistic regression to estimate the effect of the CDSS on process and patient outcomes. As this is an observational study, we will also conduct propensity score matching (1:1 matching) of high-risk patients between the preintervention and intervention phases using the greedy nearest neighbor algorithm [34,35]. See Multimedia Appendix 5 [34,35] for more information about the quantitative methods used in this study.

### Methods for Study Aim 2: Explore PREVENT’s Reach and Adoption by the Homecare Admission Staff and Describe the Tool’s Implementation During Homecare Admission

To explore PREVENT’s reach and adoption of the proposed study, we will match qualitative and quantitative analyses of the collected data to gain an in-depth understanding of the topic. Table 1 provides a description of the RE-AIM aspects, specific questions, analytical methods, and data sources for these analyses.

#### Think-Aloud Method

Think-aloud methodology is a standard approach to elicit data about cognitive reasoning that occurs during a problem-solving task [24,36]. The think-aloud method will help answer questions about operational support (ie, 11-12, 15-16, and 18), as specified in Table 1. This methodology will be implemented to observe patient admission in a simulated environment with 20 comprehensive and diverse case scenarios of patient admissions (10 high-priority cases and 10 low or medium-priority cases). See Multimedia Appendix 6 [24,36] for more information about the think-aloud methodology.

#### Qualitative Analysis Method

Thematic analysis is a qualitative descriptive approach for identifying, analyzing, and reporting themes within data [37,38]. Researchers will use this approach to analyze data collected during the postintervention interviews and think-aloud simulations. The study research assistant will transcribe each interview into a text file. A different member of the study team will validate portions of transcriptions (20-30%) for quality. We will use qualitative analysis software (NVivio [39]) to implement the analysis. See Multimedia Appendix 7 [37-40] for more information.

#### Mixed Methods Analysis

This mixed methods analysis will match qualitative (for exploring the PREVENT’s reach and adoption) and quantitative (to evaluate the effects of PREVENT on process and patient outcomes) findings to provide context so as to gain an in-depth understanding of our experimental results. The qualitative findings will supplement the quantitative findings. For example, to understand aspects of CDSS implementation related to PREVENT’s use and user satisfaction, we will match findings from qualitative questions #9 and 10 (Table 1) with findings from quantitative questions #7 and 8. Similarly, to understand aspects of CDSS adoption related to setting characteristics and strategic alignment of PREVENT with the setting’s goals, we will match the findings from questions #5 and 6.

## Results

The study research protocol was approved by the institutional review board in October 2019. Currently, the study team is working on integrating the PREVENT CDSS into hospital and homecare EHRs. The study team is also analyzing information on patients who are currently being admitted to the VNSNY to create a framework for statistical analysis of this study’s findings. These activities are necessary to conduct study aims 1 and 2. Data collection is planned to start in early 2021.

## Discussion

### Principal Findings

In this study, we introduced a rigorous methodology for evaluating the implementation of an innovative CDSS, PREVENT, which was developed to assist in determining which patients should be prioritized for the first homecare nursing visit [18] (more details on the PREVENT tool are presented in Multimedia Appendix 1). This methodology was built on the RE-AIM framework and mixed methods approaches, incorporating homecare admission staff interviews, think-aloud simulations [24], and analysis of staffing and other relevant data. By following the methodology’s steps, we will be able to explore the reach and adoption of PREVENT by the homecare admission staff; describe the implementation phase; identify the potential barriers for implementation; and improve the perceived value (usefulness) and familiarity (ease of use) of PREVENT from the schedulers’, clinicians’, and administrative staffs’ perspectives. In addition, by exploring the technical infrastructure and the process of clinician decision making, we will be able to adjust the workflow and smoothly integrate PREVENT with the EHR system for automated computation of the first homecare nursing visit priority for individual patients.

Overall, our approach for the evaluation of PREVENT as a CDSS implementation consists of 3 major phases: preintervention, intervention, and postintervention phases. It is crucial to measure the efficacy of the CDSS in improving health care outcomes using controlled experimental methods. In the preintervention phase, we will identify the eligible staff for exposure to the CDSS and conduct training to educate them about using the CDSS. The research team will also explore and evaluate the existing infrastructure and workflow of decision making and prepare a rigorous plan for the integration of the CDSS with the clinical workflow.

In the intervention phase, the trained admission staff will be exposed to the CDSS intervention. Monitoring and tracking the staff’s use of the CDSS recommendations and the extent to which the recommendations are overridden is essential to develop appropriate mechanisms to measure CDSS usability and adjust the infrastructure and workflow to meet the needs of administrative staff.

For the postintervention phase, the core step is to evaluate the implementation of the CDSS using appropriate instruments and tools to generate a clear picture of the barriers and facilitators for CDSS uptake and effectiveness. Tools such as qualitative interviews, think-aloud simulations, and End-User Computing Satisfaction Instrument will help the research team measure staff satisfaction and agreement with the CDSS and perceived CDSS usability and ease of use. Other important factors to be evaluated in this phase are access to resources and adjustment of workflow and technical infrastructure.

### Strengths, Limitations, and Alternatives to the Methodology

We considered several options for the study design. We could have used a stepped wedge cluster randomized trial [41]. One prerequisite for such a design is the ability to randomize the intervention among clearly defined clusters of hospital units or homecare agencies. In this study, homecare admission staff consists of 3 types of professionals (intake coordinators, clinical associate managers, and schedulers), some of whom work together on a constant basis in the same office. Thus, isolating selected admission staff is challenging, with a high risk of contamination.

Another option was to conduct a randomized controlled trial of PREVENT. This study design would require randomization at the patient level such that PREVENT recommendations would be shared for half of the randomly selected patients but not the others. Similar to the stepped wedge cluster design challenge, clear randomization would be unlikely due to contamination. The proposed quasi-experimental pre-post design (with propensity score matching) is similar to our pilot efficacy study [9] and addresses the contamination challenge of the abovementioned approaches. By matching patients in the preintervention and intervention phases based on their propensity score, we will account for potential differences in these 2 patient groups, which we were not always able to do in the pilot study. This design, guided by the RE-AIM framework, is preferable for an effectiveness study of real-world CDSS implementation.

Additional study limitations include the relatively limited generalizability of study findings, as the study involves 2 hospitals in a large academic hospital system in New York City. In addition, our ability to draw causal inferences is somewhat limited in quasi-experimental pre-post study designs.

### Innovation and Impact

Our study is innovative in the seamless use of CDSS and patient-centeredness:

- Our work is focused on building and evaluating one of the first evidence-based CDSS for homecare in the United States. The majority of hospitals and many homecare agencies across the nation use some type of EHR [42]. Tools such as PREVENT are becoming increasingly important in the effort to standardize care among agencies and avoid negative patient outcomes.
- For decades, the homecare industry has promoted providing the first nursing visit close to hospital discharge as an effective strategy to prevent hospitalizations. PREVENT will provide the first CDSS in homecare to assist agencies with implementing this important intervention.
- PREVENT will help highlight unique patient characteristics to support person-centered care. Our work is intended to change the paradigm in homecare by implementing 3 out of 4 key characteristics of the homecare agency of the future recently identified by the Institute of Medicine report * The Future of Home Health Care* [43]. The 3 characteristics are the agency being *patient and person centered, seamlessly connected, *and *technology enabled.*

Study results may have the potential to (1) standardize and individualize nurse decision making by using cutting-edge technology and (2) improve patient outcomes in the understudied homecare setting.

### Conclusions and Recommendations

This manuscript presents a protocol of a CDSS PREVENT study aimed at improving the outcomes of patients admitted to homecare services. We strongly encourage other researchers who study the effects of CDSS in clinical practice to apply similar mixed qualitative and quantitative methodologies in their studies. The application of mixed methods can enable researchers to gain an in-depth understanding of the complex socio-technological aspects of CDSS use in clinical practice. In turn, such comprehensive understanding can improve long-term effective use of CDSS in clinical settings.

## Appendix

Multimedia Appendix 1 Development of the Priority for the First Nursing Visit Tool.

Multimedia Appendix 2 Study instruments.

Multimedia Appendix 3 Minimum detectable difference between high priority intervention group patients versus pre-intervention high priority patients.

Multimedia Appendix 4 Rehospitalization data.

Multimedia Appendix 5 Quantitative analysis methods.

Multimedia Appendix 6 Think-aloud methodology.

Multimedia Appendix 7 Qualitative analysis methods.

Multimedia Appendix 8 Peer-Review Reports from National Institute of Health.

## Abbreviations

CDSS: clinical decision support system
EHR: electronic health record
MAPLe: Method for Assigning Priority Levels
NYP: New York-Presbyterian
OASIS: Outcome and Assessment Information Set
PREVENT: Priority for the First Nursing Visit Tool
RE-AIM: Reach, Effectiveness, Adoption, Implementation, and Maintenance
RN: registered nurses
VNSNY: Visiting Nurse Service of New York
