# Supplementary material for: Improving Patient Prioritization During Hospital-Homecare Transition: Protocol for a Mixed Methods Study of a Clinical Decision Support Tool Implementation
Source: JMIR Res Protoc. 2021 Jan 22;10(1):e20184. doi: 10.2196/20184 (PMC7864770; doi:10.2196/20184)
Supplement: Multimedia Appendix 1 [file resprot_v10i1e20184_app1.docx]

# Multimedia Appendix 1: Development of the PREVENT tool

Development of the PREVENT was guided by a strong theoretical model, namely the Transitions theory [17]. Also, we used our established methodology for eliciting expert knowledge to build CDSS[18]. Adjusted logistic regression was fit to predict nurses’ decisions on patient visit priority. The total study sample included 670 patients, with 70% used for model development and 30% for model testing. The resulting prediction model achieved an Area under the Curve (AUC) of 75.9 when validated on the model testing sample. Models with AUC > .70 are considered valid [40].

Five patient risk factors were identified as significant predictors of patient's priority for the first homecare nursing visit: (a) Presence of wounds (either surgical or pressure ulcers); (b) a documented comorbid condition of depression; (c) need for assistive equipment, assistive person, or both for toileting; (d) number of medications; and (e) number of comorbid conditions. These risk factors are also commonly identified in the literature as major contributors to rehospitalizations from homecare[41,42].

Each risk factor was assigned a specific score based on the logistic regression weights. For instance, for a wound (e.g., pressure ulcer, vascular ulcer), the patient received a score of 15 points. For each additional co‐morbid condition, one point was added to the final score. Summing the scores for the factors generated a cumulative score. The optimal cut‐off point was established based on the regression model performance statistics[16], indicating that patients with a score greater than 26 points are a high priority for the first homecare nursing visit.
