# Supplementary material for: Improving Patient Prioritization During Hospital-Homecare Transition: Protocol for a Mixed Methods Study of a Clinical Decision Support Tool Implementation
Source: JMIR Res Protoc. 2021 Jan 22;10(1):e20184. doi: 10.2196/20184 (PMC7864770; doi:10.2196/20184)
Supplement: Multimedia Appendix 2 [file resprot_v10i1e20184_app2.docx]

**Multimedia** **Appendix 2: Study instruments**

The “post-intervention phase interview guide” will include a separate section with 3-4 questions about cases where admission staff disagreed with PREVENT decisions. These questions (e.g., “Compared to PREVENT factors, what additional factors made this patient a priority in your view?”) will be asked over the phone (next day follow-up) of the admission staff (schedulers and clinical field managers, when necessary) that was involved in admitting patients sooner or later than suggested by the PREVENT tool. We will also examine free text comments about the reasons not to provide priority visits indicated within VNSNY EHR in the intervention and post-intervention phases. The study team will pilot test and revise each of the interview questions and guides until deemed final. The experienced interviewers on the study team will provide the interviewers in-depth training on the methodology and interview techniques before they conduct the interviews and observations.

The End-User Computing Satisfaction Instrument [28–30] will be used to quantitatively measure satisfaction. The 12 item instrument measures the concepts of accuracy (2 questions), content (4 questions), format (2 questions), ease of use (2 questions), timeliness (2 questions) and overall end-user satisfaction (total score). The instrument has been successfully used to evaluate all types of applications, including decision support, and takes less than 10 minutes to administer. Satisfaction ratings for each concept are scored as 1 (almost never); 2 (some of the time); 3 (about half the time); 4 (most of the time); 5 (almost always). Minimum score is 12; maximum 60. A score of 54 corresponds to the 70th percentile. 57 = 80th and 59 = 90[28–30]. Results will also be reported by percentages of respondents by Likert scale level and domain, as well as grouped by percent favorable and unfavorable. Any concept scoring less than the 70th percentile from either user group will guide future tool revision and an average overall score of 75th percentile or higher will be deemed as an acceptable provider response to the CDSS.
