# Supplementary material for: Improving Patient Prioritization During Hospital-Homecare Transition: Protocol for a Mixed Methods Study of a Clinical Decision Support Tool Implementation
Source: JMIR Res Protoc. 2021 Jan 22;10(1):e20184. doi: 10.2196/20184 (PMC7864770; doi:10.2196/20184)
Supplement: Multimedia Appendix 3 [file resprot_v10i1e20184_app3.docx]

**Multimedia Appendix 3: Sample Size**

Table 1 in this appendix presents the minimum detectable difference between high priority intervention group patients (i.e., those high priority patients whose PREVENT score was shared with intake personnel) versus pre-intervention high priority patients, with separate multivariate logistic regression models of rehospitalization. We present two scenarios where we assume square multiple correlations (SMC) of 0.20 and 0.30. As this table shows, we conservatively expect to detect smaller differences in hospital readmission rates for high priority patients than the rates found in the pilot study at 90% power, with the proposed number of referrals in the pre- and intervention periods after adjusting for patient characteristics that might be also associated with rehospitalizations.

Table 1-appendix C: Sample size calculations

|  | Rehospitalizations for Patients Classified as “High Risk” by PREVENT (N=2094) | | | | | | | |
| --- | --- | --- | --- | --- | --- | --- | --- | --- |
|  | SMC = 0.20 | | | | SMC = 0.30 | | | |
| Power | Pre-intervention | Intervention | Difference | OR | Pre-intervention | Intervention | Difference | OR |
| 0.80 | 0.211 | 0.149 | 0.062 | 0.655 | 0.211 | 0.145 | 0.066 | 0.634 |
| 0.85 | 0.211 | 0.145 | 0.066 | 0.634 | 0.211 | 0.141 | 0.070 | 0.612 |
| 0.90 | 0.211 | 0.140 | 0.071 | 0.609 | 0.211 | 0.135 | 0.076 | 0.586 |
