# Supplementary material for: Improving Patient Prioritization During Hospital-Homecare Transition: Protocol for a Mixed Methods Study of a Clinical Decision Support Tool Implementation
Source: JMIR Res Protoc. 2021 Jan 22;10(1):e20184. doi: 10.2196/20184 (PMC7864770; doi:10.2196/20184)
Supplement: Multimedia Appendix 4 [file resprot_v10i1e20184_app4.docx]

## Multimedia Appendix 4: Rehospitalization data

Rehospitalization data: Most often when patients are rehospitalized, they return to the same facility as the initial hospitalization: In this study it would be the NYP system. We will have access to this data. In order to confirm all potential rehospitalizations and ED visits we will use data from a Regional Health Information Organization (RHIO), covering New York City, Nassau and Suffolk Counties . RHIOs in New York State cover different areas from Buffalo to New York City. The RHIOs allow health care providers to electronically track admissions, discharges and transfers to and from varying health care organizations within the State. The RHIO that the study team will collaborate with will cross reference VNSNY case study patients across the other RHIOs in New York to be certain we are capturing all hospitalizations following homecare discharge. The RHIO will provide dates for all acute care utilization or ED use within the study period. This check will be used to estimate the rate of patient rehospitalizations or ED use within 60 days since the initial hospital discharge (at the time of study enrollment). For each patient (and when available), we will calculate the time to first rehospitalization or ED use (in days), and whether patients were rehospitalized or admitted to ED within 30 and 60 days since the initial hospital discharge (at the time of study enrollment).
