# Supplementary material for: Improving Patient Prioritization During Hospital-Homecare Transition: Protocol for a Mixed Methods Study of a Clinical Decision Support Tool Implementation
Source: JMIR Res Protoc. 2021 Jan 22;10(1):e20184. doi: 10.2196/20184 (PMC7864770; doi:10.2196/20184)
Supplement: Multimedia Appendix 5 [file resprot_v10i1e20184_app5.docx]

**Multimedia Appendix 5: Quantitative analysis methods**

We will use survival analysis methods and logistic regression to estimate the effect of the CDSS on process and patient outcomes. First, we will assess if there are any differences in socio-demographic and clinical characteristics between the patients in the pre-intervention and intervention phases, as well as between the two hospitals. We will estimate the time from hospital discharge to the first home care visit, and rates of and time to hospital readmission for high and low/medium priority patients. We will adjust these estimates for socio-demographic and clinical characteristics, and clustering at the hospital level.

Because this is an observational study, we will also conduct propensity score matching (1:1 matching) of high risk patients between the pre- and intervention phases using the Greedy nearest neighbor algorithm [31,32]. We will assess whether matching resulted in balanced groups by examining the standardized differences in means. If any variables remain unbalanced after matching, we will adjust for those variables when comparing the outcomes of interest. To identify any potential bias introduced by their exclusion from the analysis sample, we will describe the group from which we were not able to find a match and compare it to the matched group. We will also conduct a sensitivity analysis and compare the survival and regression models results with and without propensity score matching. This analysis is especially important for patients for whom we might not be able to find a match. We will apply logistic regression to assess PREVENT’s effect on the timing of the first homecare visit and rate of rehospitalizations. We will use survival analysis methods to assess PREVENT’s effect on the time to rehospitalization.
