# Supplementary material for: Improving Patient Prioritization During Hospital-Homecare Transition: Protocol for a Mixed Methods Study of a Clinical Decision Support Tool Implementation
Source: JMIR Res Protoc. 2021 Jan 22;10(1):e20184. doi: 10.2196/20184 (PMC7864770; doi:10.2196/20184)
Supplement: Multimedia Appendix 6 [file resprot_v10i1e20184_app6.docx]

## Multimedia Appendix 6: Think-aloud methodology

Think-aloud methodology is a standard approach to elicit data about cognitive reasoning which occurs during a problem solving task[21,33]. The think-aloud method will help answer questions 11-12, 15-16, and 18 of this study, as specified in Table 1. The methodology will be implemented to observe patient admission in a simulated environment with 20 comprehensive and diverse case scenarios of patient admissions (10 high priority cases and 10 low/medium priority cases). These cases will present the same amount of information as often available to the admission staff per role. Participants will be asked to verbalize their actions and decision making performing their regular duties. We expect to observe 20 admission staff members at a convenient and quiet location next to their offices. During observations, two members of the research team will be present during use of the modified think-aloud protocol. Each observation will last approximately 10 minutes per admission. We will observe 5 simulated admissions per observation session (one session per participant). At the end of the observations, participants will be asked a series of follow-up questions to elicit general attitudes and descriptive details about patient admission, CDSS usefulness and usability, and CDSS impact on workflow. All interviews will be audio recorded. Both observers will take field notes.
