# Supplementary material for: Improving Patient Prioritization During Hospital-Homecare Transition: Protocol for a Mixed Methods Study of a Clinical Decision Support Tool Implementation
Source: JMIR Res Protoc. 2021 Jan 22;10(1):e20184. doi: 10.2196/20184 (PMC7864770; doi:10.2196/20184)
Supplement: Multimedia Appendix 7 [file resprot_v10i1e20184_app7.docx]

## Multimedia Appendix 7: Qualitative analysis methods

Thematic analysis is a qualitative descriptive approach for identifying, analyzing and reporting themes within data [34,35]. Researchers will use this approach to analyze data collected during the post-intervention interviews and think-aloud simulations. The study research assistant will transcribe each interview into a text file. A different member of the study team will validate portions of transcriptions (20-30%) for quality. We will use qualitative analysis software (NVivio[36]) to implement the analysis. The six analyses phases are: (1) Familiarization with the data - the study team will acquaint themselves with the interviews by listening to the recordings, reading the transcribed data, and documenting initial ideas. (2) Generating initial codes – researchers will create an initial coding scheme based on the study's conceptual framework, RE-AIM. (3) Data coding - two researchers trained in qualitative analysis will independently code the transcribed interviews. They will assign codes to informative features of the data (phrases, sentences, or paragraphs). Inter-coder reliability will be assured by dual coding of the first several interviews. After each interview, the two coders will review the coding for similarities and variations by comparing the level of agreement and discuss discrepancies. Once inter-coder reliability exceeds 90% agreement, each researcher separately will continue to code all remaining interviews. The two coders will discuss new codes that emerge and add the new codes to the coding scheme, when necessary. (4) Searching for themes – researchers will collate codes into potential themes, so as to organize the data relevant to each potential theme. The team will evaluate themes so as to estimate their fit in relation to the coded extracts and the entire data set. (5) Defining and naming themes – researchers will define and further refine themes that emerge. For each theme, researchers will identify the “essence‟ of what each theme is about and determine what aspect of the data each theme captures. Doing so will help to generate clear definitions and names for each theme. (6) Producing the report – researchers will generate a final report with a selection of vivid and compelling interview quotes. Study themes will relate back to the research question and literature review.

The study team will maintain methodological rigor of the thematic data analyses with an audit trail and periodic debriefing. Reliability will be measured via consistency of interpretation and coding of the qualitative data[43]. An audit trail will include process and analytic memos, and coding books to support the credibility and reproducibility of the results.
