# Supplementary material for: Improving Patient Prioritization During Hospital-Homecare Transition: Protocol for a Mixed Methods Study of a Clinical Decision Support Tool Implementation
Source: JMIR Res Protoc. 2021 Jan 22;10(1):e20184. doi: 10.2196/20184 (PMC7864770; doi:10.2196/20184)
Supplement: Multimedia Appendix 8 [file resprot_v10i1e20184_app8.pdf]

SUMMARY STATEMENT
(Privileged Communication)

PROGRAM CONTACT:
Augusto Diana
augusto.diana@nih.gov

Release Date: **06/25/2019**
Revised Date:

Application Number: **1 R01 NR018831-01**

Principal Investigator

TOPAZ, MAXIM

Applicant Organization: COLUMBIA UNIVERSITY HEALTH SCIENCES

Review Group: **DIRH**
Dissemination and Implementation Research in Health Study Section

Meeting Date: **06/12/2019**
Council: **OCT 2019**
Requested Start: **09/01/2019**
RFA/PA: **PA18-145**
PCC: **CDPAD**

Project Title: **Improving patient prioritization during hospital-homecare transition: A mixed methods study of a clinical decision support tool**
SRG Action: **Impact Score:30 Percentile:21**
Next Steps: **Visit https://grants.nih.gov/grants/next_steps.htm**
Human Subjects: **30-Human subjects involved - Certified, no SRG concerns**
Animal Subjects: **10-No live vertebrate animals involved for competing appl.**
Gender: **1A-Both genders, scientifically acceptable**
Minority: **1A-Minorities and non-minorities, scientifically acceptable**
Age: **3A-No children included, scientifically acceptable**

Project Year	Direct Costs Requested	Estimated Total Cost
1	461,948	763,244
2	480,070	793,186
3	476,763	787,722
TOTAL	1,418,781	2,344,152

ADMINISTRATIVE BUDGET NOTE: The budget shown is the requested budget and has not been adjusted to reflect any recommendations made by reviewers. If an award is planned, the costs will be calculated by Institute grants management staff based on the recommendations outlined below in the COMMITTEE BUDGET RECOMMENDATIONS section.
NEW INVESTIGATOR

1R01NR018831-01 Topaz, Maxim

NEW INVESTIGATOR

RESUME AND SUMMARY OF DISCUSSION: This application proposes to test the efficacy of a clinical decision support system, Priority for the First Nursing Visit Tool (PREVENT) and test its effectiveness among patients to reduce rehospitalization rates after patients are released from the hospital. This is a highly significant study, as it addresses hospital to homecare transition and aims to reduce rehospitalization. There is strong rigor of prior research which shows immediate home visits after hospital release can improve outcomes. This is a strong team of investigators with relevant experience in clinical decision-making informatics and home health research, and a strong environment. This is an innovative project, as it is a technology-enabled home health care intervention and clinical decision support in the homecare setting. Several strengths in the approach were noted, including a thorough description of the qualitative and quantitative methods, a descriptive overview of the evaluation plan, and good use of the RE-AIM model. However, reviewers felt the approach had moderate scientific rigor and highlighted several weaknesses. There is no mention of a theoretical framework and workflow integration was not clearly addressed. Reviewers wondered how participants will be recruited for the qualitative portion of the study and are unsure why the two hospitals mentioned were chosen. In addition, sex as a biological variable was not adequately addressed. Overall, reviewers agreed the study is innovative and highly significant and could have a high impact on rehospitalization among patients released from hospital.

DESCRIPTION (provided by applicant): Improving patient prioritization during hospital-homecare transition: A mixed methods study of a clinical decision support tool. Each year, more than 5 million patients are admitted to the approximately 12,000 homecare agencies across the United States. About 20% of homecare patients are rehospitalized during the homecare episode, with as many as 68% of these rehospitalizations occurring within the first two weeks of services. A significant portion of these rehospitalizations may be prevented by timely and appropriately targeted allocation of homecare services. The first homecare nursing visit is one of the most critical steps of the homecare episode. This visit includes an examination of the home environment, a discussion regarding whether a caregiver is present, an assessment of the patient capacity for self-care, and medication reconciliation. A unique care plan is created based on this evaluation of the patient's needs. Hence, appropriate timing of the first visit is crucial, especially for patients with urgent healthcare needs. However, nurses often have very limited and inaccurate information about incoming patients and patient priority decisions vary significantly between nurses. We developed an innovative decision support tool called "Priority for the First Nursing Visit Tool" (PREVENT) to assist nurses in prioritizing patients in need of immediate first homecare nursing visits. In a recent efficacy pilot study of PREVENT, high-risk patients received their first homecare nursing visit a half day sooner as compared to the control group, and 60-day rehospitalizations decreased by almost half as compared to the control group. The proposed study assembles a strong interdisciplinary team of experts in health informatics, nursing, homecare, and sociotechnical disciplines to evaluate PREVENT in a pre-post intervention effectiveness study. Specifically, the study aims are: Aim 1) Evaluate the effectiveness of the PREVENT tool on process and patient outcomes. Using survival analysis and logistic regression with propensity score matching we will test the following hypotheses: Compared to not using the tool in the pre-intervention phase, when homecare clinicians use the PREVENT tool, high risk patients in the intervention phase will: a) receive more timely first homecare visits and b) have decreased incidence of rehospitalization and have decreased emergency department (ED) use within 60 days. Aim 2) Explore PREVENT's reach and adoption by the homecare admission staff and describe the tool's implementation during homecare admission. Aim 2 will be assessed using mixed methods including homecare admission staff interviews, think-aloud simulations, and analysis of staffing and other relevant data. This innovative study addresses several National Institute of Nursing Research strategic priorities, such as promoting

innovation and using technology to improve health. Mixed methods will enable us to gain in-depth understanding of the complex socio-technological aspects of hospital-homecare transition.

PUBLIC HEALTH RELEVANCE: Our work is focused on building and evaluating one of the first evidence-based clinical decision support tools for homecare in the United States. Our results have the potential to standardize and individualize nursing decision making using cutting-edge technology and to improve patient outcomes in the homecare setting.

CRITIQUE 1

Significance: 1
Investigator(s): 1
Innovation: 1
Approach: 5
Environment: 1

Overall Impact: This application is from a New Investigator addresses a highly significant issue – high rates of rehospitalization among patients released from hospital to home health care. The research team proposes to test the efficacy of PREVENT (Priority for the First Nursing Visit Tool), which in a small pilot study suggested effectiveness in cutting time to first home health visit for “high risk” patients and reducing rehospitalization rates. The current study seeks to test effectiveness in a larger sample of patients and examine implementation in partnership with the Visiting Nurses Association of New York. The proposal presents both a strong scientific premise and high rigor of prior research. The investigative team is strong, convening investigators with impressive backgrounds in home health research, informatics, and clinical decision making. The proposed intervention is highly innovative and consistent with national recommendations promoting person-centered, seamless, and technology-enabled home health care interventions. The major weakness of the proposal is in the methodological approach (moderate scientific rigor), which in this reviewer’s opinion misapplies the RE-AIM implementation framework and does not adequately justify selection of just two hospitals within the same hospital system to examine implementation. Sex as a biological variable is not adequately addressed. However, there were also a number of strengths in the Approach section. Overall, this is viewed as a highly significant and innovative proposal that requires additional attention to scientific rigor.

1. Significance:

Strengths

- Major: The proposed research is highly significant. Almost 5 million people receive homecare in the U.S. and these numbers are expected to increase substantially over the next 15 years. A majority of these patients (70%) are admitted to homecare from hospitals. The complexity of these patients and the challenges of hospital-to-home transition contributes to a 20% rehospitalization rate, with a majority of returns to hospital occurring in the first two weeks of homecare. There is rigorous prior research, some conducted by members of the study team, showing that certain clinical decisions (e.g., “frontloading” home visits) can improve outcomes in some home healthcare patients.
- Major: CMS requires an initial patient assessment within 48 hours of homecare admission; however, there is currently no widely used method of prioritizing patients at highest need both for assessment and visit frontloading. PREVENT will use the investigators prior work in clinical decision support systems to automate this prioritization process, with the goal of reducing rehospitalizations. In pilot testing, this approach resulted in earlier visits for high priority patients

and cut rehospitalization by almost half. Thus, there is a strong scientific premise for the proposed work and high potential impact on public health.

Weaknesses

- None noted.

2. Investigator(s)

Strengths

- Major: PI Topaz is a New Investigator with an impressive publication record. The proposed study is an outgrowth of his prior dissertation work on patient prioritization in home health care. He was also involved in development of a now widespread system for clinical decisionmaking in post-acute care (D2S2).
- Major: Two of the Co-Is, Cato and Sockolow, have strong backgrounds in informatics, with specific concentrations in clinical decision support (Cato) and home health care informatics (Sockolow). Notably, Dr. Sockolow is also trained in mixed methods research approaches as they relate to informatics in home health.
- Major: Co-I McDonald from VNSNY has 20 years experience conducting research in home health nursing.
- Moderate: Dr. Bowles is a more senior researcher with decades of experience in home health research and clinical decision support.
- Major: Several, if not all, of the study team members have a history of collaboration.

Weaknesses

- None noted.

3. Innovation

Strengths

- Major: The proposed work contributes to testing one of the first evidence-based clinical decision support systems for homecare in the U.S. and facilitates recommendations for frontloading and person-centered care. It is consistent with Institute of Medicine recommendations that homecare be person-centered, seamless, and technology-enabled.

Weaknesses

- None noted.

4. Approach

Strengths

- Major: The overall mechanics of how the study will be carried in terms of data collection, transfer, and analysis have been carefully considered. The data sources are appropriate and match the primary and secondary study outcomes.
- Major: Both the quantitative and qualitative analytic methods and their corresponding data sources are nicely laid out in Table 2 (although as noted below their organization within the RE-AIM framework is viewed as inappropriate).
- Moderate: Both quantitative and qualitative data analysis methods are well described.

Weaknesses

- Major: The RE-AIM framework focuses on expanding the public health impact of evidence-based interventions. With that in mind, it seems an inappropriate implementation framework for this study to determine intervention efficacy and explore use of the intervention at two sites within a single hospital system. Also note that this is a framework, not a conceptual model as described in the proposal, and the proposal lacks any appropriate conceptual model.
- Major: The study will source patients from one large and one small hospital within a single hospital system. It is not clear why these two hospitals were chosen; if diversity of setting is important, it is not clear that choosing just two hospitals will provide the variability needed to fully assess barriers and facilitators to implementation.
- Minor: The investigators state that PREVENT was developed using a "strong theoretical model" but do not name this model or describe its application to the instrument.
- Moderate: Calculation of the PREVENT score from the EHR is critical to the success of the project. It would be helpful to have more information on what exactly is involved in making this happen, as integrating fields into the EHR can be challenging.
- Moderate: More information on what is included in the pre-intervention "educational sessions" for VNSNY admissions staff.
- Major: There is no specific description of Aim 2 recruitment or corresponding justification.
- Minor: It is not clear how "next day follow-up" with admissions staff is possible – how do the researchers get back information from staff in real time?
- Minor: More information on how (and with who) the qualitative questions will be piloted should be provided.
- Moderate: It is not clear who is being targeted by the end-user computing satisfaction survey.
- Moderate: The investigators state multiple times that they will "match" the quantitative and qualitative analyses, but it is not clear what is meant by this or how it will be done. Does this refer to triangulation? How exactly will this be done? Also, anticipated end products from the qualitative analysis should be specifically noted.
- Minor: Sex is listed as a covariate but its potential to serve as an important biological factor is not addressed.

5. Environment

Strengths

- Major: Strong environment. This is a collaboration between Columbia University, Drexel University, and University of Pennsylvania nursing schools. All three are positioned to provide the investigators with the intellectual and administrative environment necessary to complete the proposed research.
- Major: Two investigators are from the Visiting Nurse Service of New York, which will play a key role in carrying out the proposed research. Notably, VNSNY is home to a Center for Home Care Policy & Research. A strong letter of support from VNSNY is provided.
- Moderate: Clinical sites are New York Presbyterian Hospital Irving Medical Center and NYP Allen Hospital, which should provide sufficient patient numbers for the study.

Weaknesses

- Major: None noted.

Study Timeline

Strengths

- Timeline is provided and is appropriate to the study aims and activities.

Weaknesses

- None.

Protections for Human Subjects

Acceptable Risks and/or Adequate Protections

Data and Safety Monitoring Plan (Applicable for Clinical Trials Only):

Acceptable

Inclusion Plans

- Sex/Gender: Distribution justified scientifically
- Race/Ethnicity: Distribution justified scientifically
- For NIH-Defined Phase III trials, Plans for valid design and analysis:
- Inclusion/Exclusion Based on Age: Distribution justified scientifically

Budget and Period of Support

Recommend as Requested

Recommended budget modifications or possible overlap identified:

CRITIQUE 2

Significance: 3
Investigator(s): 2
Innovation: 2
Approach: 2
Environment: 2

Overall Impact: The investigators propose to further investigate the use of PREVENT, a scoring system to help home health care provider organizations determine which patients need home health visits within the first 24 hours of hospital discharge. Pilot data from a pre-post study showed about a very large (~10% absolute, ~50% relative) decrease in readmissions with 60 days, but lacked rigorous adjustment for potential confounders, and did not include the qualitative analyses proposed in this follow-up. Despite the important of the topic area, the proposal's justification of the significance is somewhat undermined by citing several old findings that may have changed during the last ten years, especially with the increased focus on readmissions during this time. This magnifies a concern regarding how decreasing the time until the first visit by a mean of about 12 hours in the pilot study could have had such a large effect on readmissions. One other issue is that the approach does not consider sex as a relevant variable. However, the large effect on readmissions in the pilot data nonetheless show that the rigor of the prior research is sufficient to justify this study. When the

excellent investigative team, innovation, and environment are considered, this well-rounded proposal has moderate potential to have a large impact on home care.

1. Significance:

Strengths

- Given the widespread use of home care and predicted increase in its volume, and given the fragility of the patient population that receives home care, this work on improving its efficacy is of high significance.
- Pilot data from a pre-post study showed about a very large (~10% absolute, ~50% relative) decrease in readmissions with 60 days.

Weaknesses

- The proposal's justification of the significance is somewhat undermined by citing several old findings that may have changed during the last ten years, especially with the increased focus on readmissions during this time. This magnifies a concern regarding how decreasing the time between discharge and the first home visit by a mean of under 12 hours in the pilot study could have had such a large effect on readmissions. This enormous effect size is insufficiently recognized and acknowledged in the proposal. Further detail and discussion is necessary regarding whether this is expected based on the intervention alone, or whether it may be due to shortcomings in the pilot study. (moderate)

2. Investigator(s):

Strengths

- The investigative team is excellent, with expertise in nursing, informatics and homecare. There is representation from personnel familiar both with the intervention to be tested and the home care agency that will provide it.
- The investigators have a track record of working together and in this research area.

Weaknesses

- None noted

3. Innovation

Strengths

- Innovative to study the use of clinical decision support in the homecare setting.

Weaknesses

- None noted

4. Approach

Strengths

- Excellent comprehensive use of the RE-AIM model in guiding the evaluation.
- Figure 1 is a good overview of the steps of the evaluation plan.
- Good use of tables to show how different aspects of the evaluative plan are addressed.

- During step 2 of the pre-intervention phase, it would also be interesting to examine the extent of concordance with current pre-intervention recommendations for front-loading, as this would allow for examination of discrepancies between PREVENT and implicit judgment without the PREVENT score influencing this judgment.
- Good justification of why the pre-post design was the best available method to study this intervention, given the use of a single home health care provider and the risk of contamination.
- Commendable use of RHIO data to address the challenge of collecting utilization data across organizations.

Weaknesses

- If pilot data shows that ~75% of patients are classified as high-risk, and especially since clinicians will be encouraged not to rely on the use of PREVENT alone (which may result in even more patients being tagged as high-risk), it seems like it may be difficult for VNSNY to do >75% of visits normally planned to occur within 2 days within 1 day, since that represents just 50% of the normal time. This is addressed in the proposal itself, but not in letters from VNSNY representatives. Even if VNSNY is able to achieve this increased responsiveness, it may be concerning for generalizability to other provider organizations. (minor)
- To the extent that iNYP contains important data used to calculate PREVENT, it could result in clinicians spending less time reviewing patient's electronic health record, and simply using the PREVENT score. (minor)
- To the extent that iNYP already offers the data that will be used in PREVENT, it undermines the justification that home care providers are frequently unable to prioritize home visits because the necessary data is incorrect or missing. (minor)
- The approach does not consider sex as a relevant variable. (minor)

5. Environment

Strengths

- The size and infrastructure of VNSNY is a good setting for the first test of this intervention, although external validity to other home care provider organizations may be an issue in the future, even if this study detects an effect.

Weaknesses

- See above

Study Timeline

Strengths

-

Weaknesses

-

Protections for Human Subjects

Acceptable Risks and/or Adequate Protections

- Commendable mention of potential delay in home visits for patients deemed to be low/moderate-risk by PREVENT.

Data and Safety Monitoring Plan (Applicable for Clinical Trials Only):

Acceptable

Inclusion Plans

- Sex/Gender: Distribution justified scientifically
- Race/Ethnicity: Distribution justified scientifically
- For NIH-Defined Phase III trials, Plans for valid design and analysis:
- Inclusion/Exclusion Based on Age: Distribution justified scientifically

Budget and Period of Support

Recommend as Requested

Recommended budget modifications or possible overlap identified:

CRITIQUE 3

Significance: 3
Investigator(s): 1
Innovation: 3
Approach: 3
Environment: 1

Overall Impact: This project addresses triage decision-making of home health RNs to address patient prioritization using clinical decision support. Home care CDS is a significant opportunity and targeting the decision-maker (i.e. the RN) could improve timely care if effective. Its 2 main aims address the effectiveness of the CDS on process and patient outcomes and its reach and implementation in home care. The rigor of prior research is clearly addressed, and preliminary studies point to the acceptability and accuracy of the CDS. The approach to validating the knowledge underlying PREVENT is state of the art and CDS methods align with other studies in the field except for a low focus on usability and workflow integration. Sex as a biological variable is not described. Not clearly addressing workflow integration and usability as an aim limits the rigor and reproducibility of this proposal and makes their data collection and analysis somewhat unclear. In CDS development and testing, it is more typical to examine usability apart from implementation, but it appears to be addressed along with implementation in aim 3 instead of as a formative step prior to implementation. The relatively high # of cases classified as “high priority” in preliminary studies could create alert fatigue on the part of the home health triage team, an important unintended consequence to track. Overall, this proposal could have high impact but weaknesses in the approach reduce enthusiasm for its promise.

1. Significance:

Strengths

- Need for improved triage decision making of home health admission RNs is now and will continue to be an important health care challenge.
- Rigor of prior research and strategies for addressing them in current proposal are outlined.

Weaknesses

- Low focus on workflow integration evidence and the importance of doing so to avoid CDS unintended consequences.

2. Investigator(s):

Strengths

- Early investigator with strong publication record and experienced team.

Weaknesses

- None identified

3. Innovation:

Strengths

- Building one of the first CDSS for homecare RNs.
- Aligns with IOM recommendations and NINR strategic priorities.

Weaknesses

- Is limited in its applicability to build the science of CDS for nursing because of not addressing usability outside of satisfaction, although I do see that there are a couple of questions in the satisfaction measure that hint at usability. (minor weakness)

4. Approach:

Strengths

- Using RE-AIM is a strong way to address CDS implementation needs.
- Preliminary studies to build and validate the CDSS are useful and align with other's strategies to develop and validate predictive models to inform CDS.

Weaknesses

- Limited focus on usability and workflow integration (minor-moderate weakness)
- Design for effectiveness is of lower rigor to examine impact of CDS and could be strengthened by considering strategies to limit bias from pre-post studies although I do acknowledge justification why the design was chosen (moderate weakness).
- The relatively high # of cases classified as "high priority" in preliminary studies could create alert fatigue on the part of the home health triage team, an important unintended consequence to track (moderate weakness).

5. Environment:

Strengths

- Good resources within institution and in the clinical setting of interest.

Weaknesses

- None identified.

Study Timeline

Strengths

-

Weaknesses

-

Protections for Human Subjects

Acceptable Risks and/or Adequate Protections

- Appears to address protection adequately.

Data and Safety Monitoring Plan (Applicable for Clinical Trials Only):

Acceptable

Inclusion Plans

- Sex/Gender: Distribution justified scientifically
- Race/Ethnicity: Distribution justified scientifically
- For NIH-Defined Phase III trials, Plans for valid design and analysis: Scientifically acceptable
- Inclusion/Exclusion Based on Age: Distribution justified scientifically

Budget and Period of Support

Recommend as Requested

Recommended budget modifications or possible overlap identified:

THE FOLLOWING SECTIONS WERE PREPARED BY THE SCIENTIFIC REVIEW OFFICER TO SUMMARIZE THE OUTCOME OF DISCUSSIONS OF THE REVIEW COMMITTEE, OR REVIEWERS' WRITTEN CRITIQUES, ON THE FOLLOWING ISSUES:

PROTECTION OF HUMAN SUBJECTS: ACCEPTABLE

INCLUSION OF WOMEN PLAN: ACCEPTABLE

INCLUSION OF MINORITIES PLAN: ACCEPTABLE

INCLUSION ACROSS THE LIFESPAN PLAN: ACCEPTABLE

COMMITTEE BUDGET RECOMMENDATIONS: The budget was recommended as requested.

Footnotes for 1 R01 NR018831-01; PI Name: Topaz, Maxim

NIH has modified its policy regarding the receipt of resubmissions (amended applications). See Guide Notice NOT-OD-14-074 at http://grants.nih.gov/grants/guide/notice-files/NOT-OD-14-074.html. The impact/priority score is calculated after discussion of an application by averaging the overall scores (1-9) given by all voting reviewers on the committee and multiplying by 10. The criterion scores are submitted prior to the meeting by the individual

reviewers assigned to an application, and are not discussed specifically at the review meeting or calculated into the overall impact score. Some applications also receive a percentile ranking. For details on the review process, see http://grants.nih.gov/grants/peer_review_process.htm#scoring.

MEETING ROSTER

Dissemination and Implementation Research in Health Study Section
Healthcare Delivery and Methodologies Integrated Review Group
CENTER FOR SCIENTIFIC REVIEW
DIRH
06/12/2019 - 06/13/2019

Notice of NIH Policy to All Applicants: Meeting rosters are provided for information purposes only. Applicant investigators and institutional officials must not communicate directly with study section members about an application before or after the review. Failure to observe this policy will create a serious breach of integrity in the peer review process, and may lead to actions outlined in NOT-OD-14-073 at https://grants.nih.gov/grants/guide/notice-files/NOT-OD-14-073.html and NOT-OD-15-106 at https://grants.nih.gov/grants/guide/notice-files/NOT-OD-15-106.html, including removal of the application from immediate review.

CHAIRPERSON(S)

BARTELS, STEPHEN J, MS, MD
DIRECTOR AND MONGAN CHAIR
DEPARTMENT OF MEDICINE
MASSACHUSETTS GENERAL HOSPITAL
HARVARD UNIVERSITY
BOSTON, MA 02114

MEMBERS

AALSMA, MATTHEW, PHD
PROFESSOR
DEPARTMENT OF PEDIATRICS
SCHOOL OF MEDICINE
INDIANA UNIVERSITY
INDIANAPOLIS, IN 46203

ALHASSAN, SOFIYA, PHD *
ASSOCIATE PROFESSOR
DEPARTMENT OF KINESIOLOGY
UNIVERSITY OF MASSACHUSETTS AMHERST
AMHERST, MA 01003

ARNOLD, CONNIE L, PHD *
ASSOCIATE PROFESSOR
DEPARTMENT OF MEDICINE AND PEDIATRICS
HEALTH SCIENCES CENTER
LOUISIANA STATE UNIVERSITY
SHREVEPORT, LA 71103

ATHERLY, ADAM J, PHD *
PROFESSOR
DEPARTMENT OF MEDICINE
HEALTH SERVICES RESEARCH CENTER
THE UNIVERSITY OF VERMONT
BURLINGTON, VT 05405

BALKRISHNAN, RAJESH, PHD *
PROFESSOR
DEPARTMENT OF PUBLIC HEALTH SCIENCES
UNIVERSITY OF VIRGINIA SCHOOL OF MEDICINE
CHARLOTTESVILLE, VA 22908

BASEN-ENGQUIST, KAREN M, PHD *
PROFESSOR
DEPARTMENT OF BEHAVIORAL SCIENCE
MD ANDERSON CANCER CENTER
UNIVERSITY OF TEXAS
HOUSTON, TX 77230

BENZO, ROBERTO, MD
ASSOCIATE PROFESSOR
DIVISION OF PULMONARY AND CRITICAL CARE
MAYO CLINIC COLLEGE OF MEDICINE
ROCHESTER, MN 55902

BROWN, JEREMIAH R, PHD *
ASSOCIATE PROFESSOR
HEALTH POLICY AND CLINICAL PRACTICE
GEISEL SCHOOL OF MEDICINE
THE DARTMOUTH INSTITUTE
LEBANON, NH 03756

CARRASQUILLO, OLVEEN, MPH, MD
PROFESSOR OF MEDICINE AND PUBLIC HEALTH SCIENCES
CHIEF, DIVISION OF INTERNAL MEDICINE GERIATRICS
MILLER SCHOOL OF MEDICINE
UNIVERSITY OF MIAMI
MIAMI, FL 33101

CARSON, APRIL P, PHD
ASSOCIATE PROFESSOR AND ASSOCIATE DEAN
FOR DIVERSITY, EQUITY AND INCLUSION
DEPARTMENT OF EPIDEMIOLOGY
SCHOOL OF PUBLIC HEALTH
UNIVERSITY OF ALABAMA AT BIRMINGHAM
BIRMINGHAM, AL 35294

CHARLEBOIS, EDWIN DUNCAN, MPH, PHD *
PROFESSOR
DEPARTMENT OF MEDICINE
SCHOOL OF MEDICINE
UNIVERSITY OF CALFORNIA, SAN FRANCISCO
SAN FRANCISCO, CA 94105

CHI, BENJAMIN H, MD *
PROFESSOR
DEPARTMENT OF OBSTETRICS AND GYNECOLOGY
SCHOOL OF MEDICINE
UNIVERSITY OF NORTH CAROLINA AT CHAPEL HILL
CHAPEL HILL, NC 27514

COLON, VIVIAN, PHD *
ASSOCIATE PROFESSOR
HEALTH SERVICES ADMINISTRATION EVALUATION PROGAM
GRADUATE SCHOOL OF PUBLIC HEALTH
UNIVERSITY OF PUERTO RICO,
MEDICAL SCIENCES CAMPUS
SAN JUAN, PR 00921

DAI, HONGYING *
ASSOCIATE PROFESSOR
DEPARTMENT OF BIOSTATISTICS
UNIVERSITY OF NEBRASKA
MEDICAL CENTER
OMAHA, NE 68198--437

DUBBERT, PATRICIA M, BSN, PHD *
PROFESSOR (RETIRED)
DEPARTMENT OF PSYCHIATRY
UNIVERSITY OF ARKANSAS FOR MEDICAL SCIENCES
LITTLE ROCK, AR 72205

FASERU, BABALOLA, MBBS, MPH *
ASSOCIATE PROFESSOR
DEPARTMENT OF PREVENTIVE MEDICINE
AND PUBLIC HEALTH
UNIVERSITY OF KANSAS MEDICAL CENTER
KANSAS CITY, KS 66160

GALARRAGA, OMAR, PHD *
ASSOCIATE PROFESSOR
DEPARTMENT OF HEALTH SERVICES,
POLICY AND PRACTICE
BROWN UNIVERSITY
PROVIDENCE, RI 02912

GEPHART, SHEILA MARIA, BSN, PHD *
ASSOCIATE PROFESSOR
COMMUNITY AND SYSTEMS HEALTH SCIENCE DIVISION
COLLEGE OF NURSING
THE UNIVERSITY OF ARIZONA
ORO VALLEY, AZ 85721

GUISE, JEANNE-MARIE, MD *
PROFESSOR
DIVISION OF MATERNAL FETAL MEDICINE
OREGON HEALTH AND SCIENCE UNIVERSITY
PORTLAND, OR 97239

HAIDER, SADIA, MD *
ASSOCIATE PROFESSOR
FAMILY PLANNING AND CONTRACEPTIVE RESEARCH
DEPARTMENT OF OBSTETRICS/GYNECOLOGY
UNIVERSITY OF CHICAGO
CHICAGO, IL 60637

HANNON, MARGARET, MPH, PHD
PROFESSOR
DEPARTMENT OF HEALTH SERVICES
UNIVERSITY OF WASHINGTON
SEATTLE, WA 98105

HOUSTON, THOMAS K II, MD, MPH
PROFESSOR AND DIVISION CHIEF
DEPARTMENT OF QUANTITATIVE HEALTH SCIENCES
DIVISION OF HEALTH INFORMATICS AND
IMPLEMENTATION SCIENCE; SCHOOL OF MEDICINE
UNIVERSITY OF MASSACHUSETTS
WORCESTER, MA 01655

HOWARD, DAVID H, PHD *
PROFESSOR
DEPARTMENT OF HEALTH POLICY AND MANAGEMENT
ROLLINS SCHOOL OF PUBLIC HEALTH
EMORY UNIVERSITY
ATLANTA, GA 30322

HUDSON, SHAWNA V, PHD
PROFESSOR AND RESEARCH DIVISION CHIEF
DEPARTMENT OF FAMILY MEDICINE
AND COMMUNITY HEALTH
ROBERT WOOD JOHNSON MEDICAL SCHOOL
RUTGERS UNIVERSITY
NEW BRUNSWICK, NJ 08901

KEAN, JACOB T, PHD *
ASSOCIATE PROFESSOR
HSR&D CIEBP
RICHARD L ROUDEBUSH VA MEDICAL CENTER
ASSOCIATE PROFESSOR
UNIVERSITY OF UTAH
SALT LAKE CITY, UT 84108

KEIB, KATHERINE, PHD *
ASSISTANT PROFESSOR OF COMMUNICATION STUDIES
COMMUNICATION STUDIES
OGLETHORPE UNIVERSITY
ATLANTA, GA 30319

KEMPE, ALLISON, MD *
PROFESSOR OF PEDIATRICS AND PUBLIC HEALTH
DIRECTOR OF ACCORDS
DIRECTOR OF SCORE
SCHOOL OF MEDICINE
UNIVERSITY OF COLORADA
AUORA, CO 80045

KENNELTY, KOREY A, PHMD, PHD *
ASSISTANT PROFESSOR
DEPARTMENT OF PHARMACY PRACTICE AND SCIENCE
DIVISION OF HEALTH SERVICES RESEARCH
UNIVERSITY OF IOWA
IOWA CITY, IA 52242

KIENE, SUSAN MARIA, PHD *
PROFESSOR
DIVISION OF EPIDEMIOLOGY AND BIOSTATISTICS
SCHOOL OF PUBLIC HEALTH
SAN DIEGO STATE UNIVERSITY
SAN DIEGO, CA 92182

KLESGES, LISA M, PHD *
PROFESSOR AND FOUNDING DEAN EMERITUS
SCHOOL OF PUBLIC HEALTH
UNIVERSITY OF MEMPHIS
MEMPHIS, TN 38152

KONKLE-PARKER, DEBORAH J, BSN, MSN, PHD *
PROFESSOR
DEPARTMENT OF MEDICNE/INFECTIOUS DISEASES
SCHOOL OF NURSING
UNIVERISTY OF MISSISSIPPI MEDICAL CENTER
JACKSON, MS 39216

KUSHEL, MARGOT B, MD
PROFESSOR AND DIRECTOR
UCSF CENTER FOR VULNERABLE POPULATIONS
DIVISION OF GENERAL INTERNAL MEDICINE
ZUCKERBERG SAN FRANCISCO GENERAL HOSPITAL
UNIVERSITY OF CALIFORNIA SAN FRANCISCO
SAN FRANCISCO, CA 94143

LAMONICA, AUKJE, PHD *
ASSOCIATE PROFESSOR
DEPARTMENT OF PUBLIC HEALTH
SOUTHERN CONNECTICUT STATE UNIVERSITY
NEW HAVEN, CT 06515

MARSTELLER, JILL ANNE, PHD *
PROFESSOR
DIVISION OF HEALTH POLICY & MANAGEMENT
JOHNS HOPKINS UNIVERSITY
BALTIMORE, MD 21205

MCLEOD, BRYCE DOUGLAS, PHD
ASSOCIATE PROFESSOR
DEPARTMENT OF PSYCHOLOGY
VIRGINIA COMMONWEALTH UNIVERSITY
RICHMOND, VA 23284

MILLER, DAVID P, MD *
PROFESSOR
DEPARTMENT OF MEDICINE AND PUBLIC HEALTH SCIENCES
WAKE FOREST SCHOOL OF MEDICINE
WINSTON-SALEM, NC 27157

PALINKAS, LAWRENCE A, PHD
PROFESSOR AND CHAIR
DEPARTMENT OF CHILDREN, YOUTH AND FAMILIES
SUZZANE DWORAK-PECK SCHOOL OF SOCIAL WORK
UNIVERSITY OF SOUTHERN CALIFORNIA
LOS ANGELES, CA 90089

PATTERSON SILVER WOLF, DAVID ALLEN, PHD *
ASSOCIATE PROFESSOR
BROWN SCHOOL OF SOCIAL WORK
WASHINGTON UNIVERSITY AT ST LOUIS
ST. LOUIS, MO 63130

PEVNICK, JOSHUA M, MD *
ASSISTANT PROFESSOR
DEPARTMENT OF MEDICINE
DIVISION OF GENERAL INTERNAL MEDICINE AND DEPARTMENT OF BIOMEDICAL SCIENCES
CEDAR-SINAI MEDICAL CENTER
LOS ANGELES, CA 90048

POLLINI, ROBIN A, PHD
ASSOCIATE PROFESSOR
DEPARTMENT OF BEHAVIORAL MEDICINE AND PSYCHIATRY
SCHOOL OF MEDICINE
WEST VIRGINIA UNIVERSITY
MORGANTOWN, WV 26506

QUANBECK, ANDREW, PHD *
ASSISTANT PROFESSOR
DEPARTMENT OF FAMILY MEDICINE & COMMUNITY HEALTH
UNIVERSITY OF WISCONSIN-MADISON
MADISON, WI 53705

SARPONG, DANIEL F, PHD
PROFESSOR OF BIOSTATISTICS, ENDOWED CHAIR AND DIRECTOR
CENTER FOR MINORITY HEALTH AND HEALTH DISPARITIES RESEARCH AND EDUCATION
COLLEGE OF PHARMACY
XAVIER UNIVERSITY
NEW ORLEANS, LA 70125

WHEELER, DARRELL P, PHD *
PROVOST AND SENIOR VICE PRESIDENT FOR ACADEMIC AFFAIRS
IONA COLLEGE
NEW ROCHELLE, NY 10801

WILLIAMS, LOVORIA, PHD *
ASSOCIATE PROFESSOR
COLLEGE OF NURSING
UNIVERSITY OF KENTUCKY
LEXINGTON, KY 40536

MAIL REVIEWER(S)

WOJDYNSKI, BARTOSZ, PHD
ASSISTANT PROFESSOR AND DIRECTOR
DIGITAL MEDIA ATTENTION AND COGNITION (DMAC) LAB
DEPARTMENT OF JOURNALISM
GRADY COLLEGE OF JOURNALISM AND MASS COMMUNICATION
UNIVERSITY OF GEORGIA
ATHENS, GA 30602

SCIENTIFIC REVIEW OFFICER

FERGUSON, YVONNE OWENS, PHD
SCIENTIFIC REVIEW OFFICER
CENTER FOR SCIENTIFIC REVIEW
NATIONAL INSTITUTES OF HEALTH
BETHESDA, MD 20892

EXTRAMURAL SUPPORT ASSISTANT

BUTLER, SEAN
LEAD - EXTRAMURAL SUPPORT ASSISTANT
CENTER FOR SCIENTIFIC REVIEW
NATIONAL INSTITUTE OF HEALTH
BETHESDA, MD 20892

* Temporary Member. For grant applications, temporary members may participate in the entire meeting or may review only selected applications as needed.

Consultants are required to absent themselves from the room during the review of any application if their presence would constitute or appear to constitute a conflict of interest.
